# Compound 48/80 increases murine bladder wall compliance independent of mast cells

**DOI:** 10.1038/s41598-023-27897-6

**Published:** 2023-01-12

**Authors:** Pragya Saxena, Eli Broemer, Gerald M. Herrera, Gerald C. Mingin, Sara Roccabianca, Nathan R. Tykocki

**Affiliations:** 1 Department of Pharmacology and Toxicology, Michigan State University College of Osteopathic Medicine, East Lansing, MI USA; 2 Department of Mechanical Engineering, Michigan State University, East Lansing, MI USA; 3 Department of Pharmacology, University of Vermont, Burlington, VT USA; 4 Division of Urology, University of Vermont Larner College of Medicine, Burlington, VT USA

**Keywords:** Bladder, Physiology

## Abstract

A balance between stiffness and compliance is essential to normal bladder function, and changes in the mechanical properties of the bladder wall occur in many bladder pathologies. These changes are often associated with the release of basic secretagogues that in turn drive the release of inflammatory mediators from mast cells. Mast cell degranulation by basic secretagogues is thought to occur by activating an orphan receptor, Mas-related G protein-coupled receptor B2 (Mrgprb2). We explored the effects of the putative mast cell degranulator and Mrgprb2 agonist Compound 48/80 on urinary bladder wall mechanical compliance, smooth muscle contractility, and urodynamics, and if these effects were mast cell dependent. In wild-type mice, Mrgprb2 receptor mRNA was expressed in both the urothelium and smooth muscle layers. Intravesical instillation of Compound 48/80 decreased intermicturition interval and void volume, indicative of bladder overactivity. Compound 48/80 also increased bladder compliance while simultaneously increasing the amplitude and leading slope of transient pressure events during ex vivo filling and these effects were inhibited by the Mrgprb2 antagonist QWF. Surprisingly, all effects of Compound 48/80 persisted in mast cell-deficient mice, suggesting these effects were independent of mast cells. These findings suggest that Compound 48/80 degrades extracellular matrix and increases urinary bladder smooth muscle excitability through activation of Mrgprb2 receptors located outside of mast cells. Thus, the pharmacology and physiology of Mrgprb2 in the urinary bladder is of potential interest and importance in terms of treating lower urinary tract dysfunction.

## Introduction

The urinary bladder is a highly distensible organ and is typically capable of holding a relatively large amount of urine as it undergoes deformation during filling^1,2^. Proper bladder function (i.e., storage and periodic expulsion of urine) is attributed to a balance between two mechanical properties of bladder tissue itself: stiffness and elasticity^3,4^. Bladder wall stiffness refers to the extent to which bladder wall can resist deformation as it fills^5^, while bladder wall elasticity refers to the ability of the wall to return to its original shape and size after deformation^6^. Both of these properties depend on the expression, interaction, and interconnection between extracellular matrix (ECM) and urinary bladder smooth muscle (UBSM) cells within the bladder wall^7–9^.

The bladder ECM consists primarily of collagen and elastin fibers, which change in orientation and conformation to accommodate increasing urine volumes during filling^10,11^. Both Type I and Type III collagen fibers are widely distributed in the bladder where they surround the smooth muscle cells and provide tensile strength to the bladder wall through complex coiling^10,12^. Elastic fibers present in all layers of the bladder wall allow the bladder to recoil to its original shape after emptying^13,14^. Interactions between the UBSM and the ECM control both basal smooth muscle tone as well as non-voiding transient pressure events that drive the majority of sensory outflow to the central nervous system^15^. Because the stiffness and elasticity of the bladder wall tissue is imparted by properties of both UBSM and ECM, altered muscle tone and collagen content are present in many bladder pathologies^16^.

Lower urinary tract symptoms (LUTS), including frequency, urgency and urge incontinence, are often associated with both an alteration in clinical compliance as well as bladder inflammation^17–19^. The pathogenesis of inflammatory bladder remodeling is also associated with the degranulation of mast cells present within the bladder wall^20,21^. Basic secretagogues, such as Substance P and Compound 48/80, can rapidly degranulate these mast cells to secrete inflammatory mediators^22^. These cationic compounds are thought to initiate IgE-independent mast cell degranulation by binding to the orphan receptor Mas-related G protein-coupled receptor-B2 (Mrgprb2)^23^. Mouse Mrgprb2 receptors and their human ortholog (MRGPRX2) are implicated in nociception and inflammation in other organ systems^24^. However, the function of Mrgprb2 in the bladder is unexplored.

In this study, we used Compound 48/80 to determine if bladder mast cell activation altered bladder wall mechanical compliance during filling. We discovered that Compound 48/80 increases smooth muscle contractility while paradoxically increasing compliance in C57Bl/6J mice. The effects of Compound 48/80 on wall compliance and contractility were inhibited by the Mrgprb2 antagonist QWF, suggesting the effects are indeed mediated by Mrgprb2 activation. Surprisingly, these effects also persisted in mast cell-deficient (*C-kit*^*W-sh*^) mice, suggesting Mrgprb2 is expressed outside of mast cells in the urinary bladder. This conclusion is further reinforced by our finding that Mrgprb2 mRNA expression was similar in bladders from mast cell deficient as well as wild-type mice. Collectively, our results suggest that Compound 48/80 increases bladder wall compliance and UBSM excitability through activation of Mrgprb2 receptors expressed outside of mast cells. Mrgprb2 receptors may thus have an important physiological and pathological function in the urinary bladder.

## Materials and methods

### Animal care and use

All animal procedures followed institutional guidelines and were approved by the Institutional Animal Care and Use Committees (IACUC) of Michigan State University (NIH Assurance D16-0054). All procedures followed the recommendations in the ARRIVE guidelines. Male C57Bl/6 mice and male mast cell-deficient (*C-kit*^*W-sh*^) mice (9–12 weeks old; Jackson Laboratory, Bar Harbor, ME USA) were group housed in a temperature- and humidity-controlled environment with a 12-h light/dark cycle and ad libitum access to standard chow and water. Mice were euthanized by intraperitoneal injection of pentobarbital (> 150 mg/kg) followed by decapitation prior to all experimental procedures.

### Ex vivo whole bladder filling using the pentaplanar reflected image macroscopy (PRIM) System

**Figure 1:**
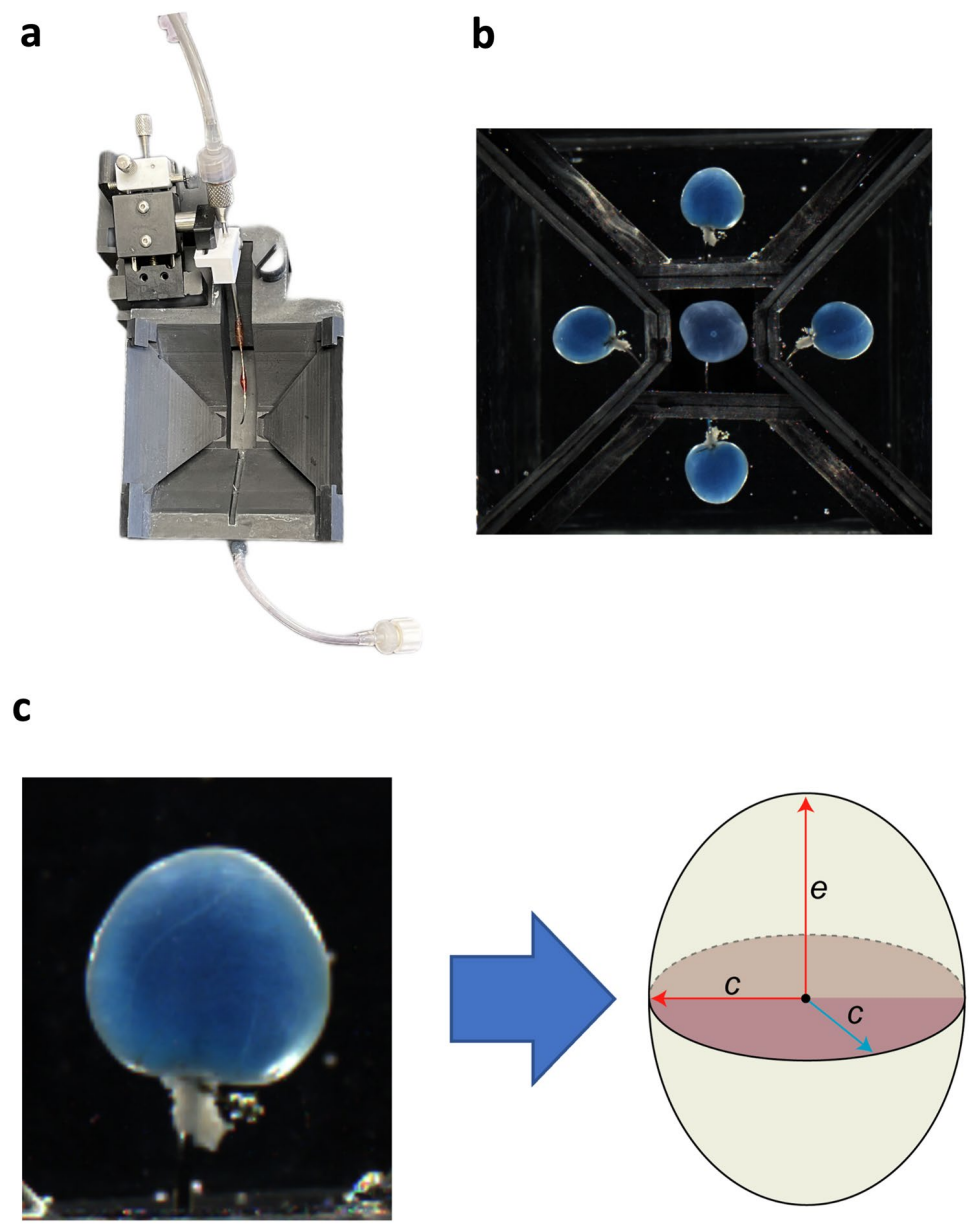
Pentaplanar reflected image macroscopy system. (**a**) Top view of Novel pentaplanar reflected image macroscopy (PRIM) system chamber. The mirrors were removed to make the cannula visible. (**b**) Single frame of ex vivo bladder filling video recording, showing all 5 visual planes recorded in the PRIM chamber. These images were used to calculate area and thickness of the bladder wall. (**c**) the bladder was modeled as an ellipsoid with radii c and e. This assumption was made to accurately calculate the geometrical parameters and bladder wall volume used for later determination of stress and stretch during filling.

Urinary bladders were dissected and placed in ice-cold Ca^2+^-free HEPES dissection buffer containing (in mM): NaCl (134), KCl (6), MgCl_2_ (1.2), HEPES (10) and glucose (7); pH = 7.4. Tissues were cleaned of connective tissue, pinned in a dissecting dish, and ureters tied off with 4-0 suture. The bladder was then cannulated through the urethra in the Pentaplanar Reflected Image Macroscopy (PRIM) chamber. The PRIM system consists of an imaging chamber (Fig. 1a) with a curved cannula and 4 mirrors fixed at 45° angles. A digital camera (DMK33UX250; The Imaging Source, Charlotte NC USA) was mounted above the chamber along with LED lighting to illuminate the sample. When properly aligned, 5 visual planes were in focus and able to be recorded in a single image (Fig. 1b). Images and pressure readings were recorded simultaneously at a rate of 10 Hz. Intravesical pressure was recorded via an inline pressure transducer (DYNJTRANSMF; Medline, Northfield, IL, USA) connected to the cannula.

Throughout the experiment, the PRIM chamber was recirculated with warm (37 °C) bicarbonate-buffered physiological salt solution (PSS) consisting of (mM): NaCl (119), NaHCO_3_ (24), KCl (4.7), KH_2_PO_4_ (1.2), MgCl_2_ (1.2), and CaCl_2_ (2) using a recirculating peristaltic pump. PSS was bubbled throughout the duration of the experiment with biological atmosphere gas (21% O_2_, 5% CO_2_, 74x% N_2_) to maintain pH and tissue oxygenation. Bladders were then filled through the cannula at a rate of 30 µL/min using a syringe pump until a maximum pressure of 25 mmHg was reached. To increase contrast, the infused PSS was mixed with purple food coloring. After reaching 25 mmHg, the bladder was then emptied and allowed to re-equilibrate at atmospheric pressure for 5–10 min before the next fill. Each bladder underwent 3 equilibration fills prior to 3 experimental fills. To account for variability, comparisons were made between the same three consecutive fills in each bladder: (1) the antepenultimate fill and empty cycle (control); (2) the penultimate fill and empty cycle in presence of vehicle or inhibitor; and (3) the final fill and empty cycle after the addition of agonist. As previously described, all drugs and their respective vehicles were incubated for 30 min prior to filling cycles^15^.

### Calculation of true wall compliance

Wall stress is defined as the force per unit cross-sectional area exerted tangential to the wall in response to the outward pressure exerted by the fluid inside the bladder^25^. Wall stretch is defined as a measure of deformation that can be given as change in size of the bladder with reference to its original size^25^. The urinary bladder wall was assumed to be a 3-dimensional elastic system; thus, bladder wall compliance was quantified as wall stress versus stretch. Bladder wall area and thickness were derived from the PRIM system images using ImageJ (NIH). These measurements were then used to model the bladder as an ellipsoid of radii c and e (Fig. 1c), with constant wall volume and uniform thickness, that behaves as a non-linearly expanding deformable vessel as described previously^26^. For each value of pressure, we evaluated the radius of the sphere of equivalent wall volume to the ellipsoid, and employed the Laplace equation to evaluate the spherical Cauchy stress as:

$${\sigma }_{w}=\frac{{P}_{\mathrm{ves}}\cdot r}{2\cdot t}$$

where ${P}_{\mathrm{ves}}$ is intravesical pressure, and $r$ and $t$ are the radius and thickness of the equivalent sphere in the current configuration (i.e., for $P={P}_{\mathrm{ves}}$).

The stretch was used as a measure of deformation and was calculated as:

$$\lambda =\frac{r}{{R}_{i}}$$

where ${R}_{i}$ is defined as the radius of the equivalent sphere in the initial configuration (i.e., for $P\le 1 \; \text{mmHg}$). Under the assumption of incompressibility^27^, we can substitute for the current thickness $t$ with the initial wall thickness ${(T}_{i})$ as:

$$t=\frac{{T}_{i}}{{\lambda }^{2}}$$

Both stress and stretch were plotted for each time point during ex vivo bladder filling. To summarize data from multiple experiments, stress and stretch recordings were first plotted at each corresponding pressure value in 5 mmHg increments (0–25 mmHg) using a custom MATLAB (MathWorks; Natick, MA USA) program. The program was also used to calculate wall stiffness (defined as the slope of stress/stretch curve) at 5, 10, and 25 mmHg. All curves belonging to the same group were then averaged for both stress and stretch using GraphPad Prism (GraphPad Software; San Diego, CA USA). Clinical compliance for all groups was also calculated as described previously^28^.

### Analysis of transient pressure events

Transient pressure events were measured and analyzed using LabChart Pro (ADInstruments; Sydney, Australia). The transient pressure event amplitude and leading slope were measured for all events occurring over the range of physiological voiding pressures (1–11 mmHg), as described previously^15^. To represent the effects of drug and vehicle, $\Delta$ Peak Amplitude and $\Delta$ Leading slope were calculated by subtracting their average values after exposure to drug from average control values prior to exposure to drug.

### Quantitative RT-PCR

Unless otherwise noted, all qRT-PCR primers, reagents, and equipment were obtained from Thermo-Fisher Scientific (Waltham, MA USA). Urinary bladders were dissected, cut longitudinally from urethera to dome, pinned *en face*, and denuded of urothelium by blunt dissection. Both urothelium and UBSM layers were saved separately in *RNA Later* for RNA isolation. Total RNA was isolated and purified using RNAeasy Mini Kit for whole tissues (Qiagen; Hilden, Germany) according to the manufacturer's instructions. The yield and purity of the RNA was measured photometrically using a Nanodrop 2000c. A High-Capacity cDNA Reverse Transcription kit was used to reverse transcribe RNA to first-strand cDNA in a 96-well thermocycler (Applied Biosystems). The yield and purity of resultant cDNA was also measured photometrically using a Nanodrop 2000c. Expression of target mRNAs was measured from each sample by qPCR in the QuantStudio 6 Flex Real-Time PCR System with Taqman Universal PCR Mastermix. Cycle conditions were as follows: 2 min at 50 °C, 10 min at 95 °C, 40 cycles of 15 s at 95 °C and 1 min at 60 °C. Each PCR procedure included a no-template negative control reaction. Expression of *Actb* (Mm02619580_g1), *Acta2* (Mm00725412_s1), *Upk2* (Mm00447665_m1), and *Mrgprb2* (Mm01956240_s1) were measured, using *Actb* as a reference gene.

### Conscious cystometry

Conscious cystometry was performed as described previously^29^. Briefly, mice were anaesthetized with inhaled isoflurane (1–3% in O_2_) and a lower midline abdominal incision was made to expose the urinary bladder. A polyethylene catheter (PE-10) was inserted into the dome of the urinary bladder and secured in place using a purse string suture. The bladder catheter was sealed and routed subcutaneously to the back of the neck, where it was coiled and stored in a skin pouch. Following a 3-day recovery period, the bladder catheter was exteriorized and opened. The animal was placed in a Small Animal Cystometry Lab Station (MED Associates, Georgia, VT USA) for urodynamic measurements. An in-line pressure transducer was connected between the catheter and a syringe pump. Sterile isotonic saline (0.9% NaCl; room temperature) was continuously infused into the bladder at a rate of 30 μL/min. An analytical balance beneath the wire-bottom animal cage measured void volumes during continuous cystometry. A single cystometrogram (CMG) was defined as the simultaneous recording of intravesical pressure, infused volume, and voided volume during a single filling-voiding cycle. Cystometrograms were recorded before and after intravesical infusion of Compound 48/80 (50 µg/mL). Data analysis was performed by averaging CMGs recorded from separate mice under the same conditions.

### Drugs and chemicals

QWF was obtained from Tocris Bioscience (Bristol, UK). Unless otherwise specified, Compound 48/80 and all other reagents were obtained from Sigma-Aldrich (Cleveland, OH, USA).

### Statistical analysis

For comparison of two samples of equal variance, statistical significance between groups were assessed using two-tailed, paired Student’s *t* tests ($\alpha =0.05)$. For multiple sample comparisons of equal variance, ordinary one-way ANOVA was used followed by Tukey’s post-hoc analysis to compare individual means. Calculations were performed using Excel (Microsoft Corporation; Redmond, WA, USA) or GraphPad Prism. Values are expressed as mean ± SEM. For clarity, data points from C57Bl/6 mice are represented by circles and *C-Kit*^*W-Sh*^ mice are represented by squares in all figures. Comparisons with *P* values < 0.05 were considered statistically significant. Asterisks were used to express statistical significance in figures: *P < 0.05 and **P < 0.01. Exact *P* values are stated in the results. For clarity, “N” represents the number of animals in each group. Multiple replicates from a single animal are not used or reported in this study.

## Results

### Mas-related G protein-coupled receptor B2 (Mrgprb2) receptor mRNA is expressed in urinary bladder smooth muscle and urothelium

**Figure 2:**
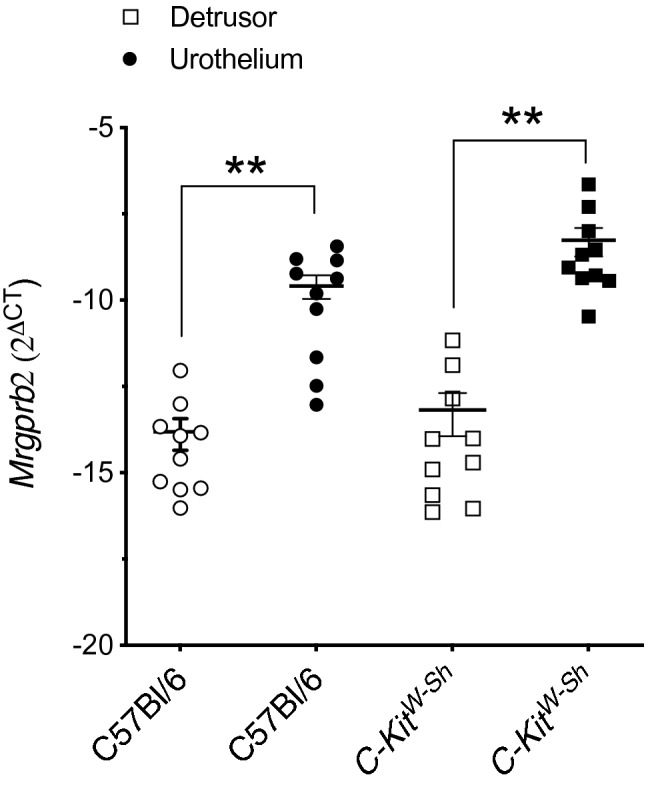
Isolated bladder tissue from both wild-type and mast cell-deficient mice express Mrgprb2 mRNA. Mrgprb2 mRNA expression in the urinary bladder smooth muscle layer and urothelial layer from both C57Bl/6 (N = 10) and *C-Kit*^*W-Sh*^ mice (N = 10), as measured by qRT-PCR. Both mouse models express Mrgprb2 in the bladder in both layers and to a similar extent [*P* = 0.442 and *P* = 0.0555 for detrusor and urothelium respectively (C57Bl/6 *vs. C-Kit*^*W-Sh*^)]. Mrgprb2 mRNA expression in the urothelium is significantly higher in both mouse models [(*P* = 0.0028 and *P* = 0.0061 for C57Bl/6 and *C-Kit*^*W-Sh*^ respectively (Detrusor vs. Urothelium)]. Data shown as Mrgprb2 expression relative to the housekeeping gene, ß-actin.

Quantitative RT-PCR was used to determine *Mrgprb2* mRNA expression in isolated urothelium and smooth muscle tissues from wild-type and *C-kit*^*W-sh*^ mice. Both layers expressed *Mrgprb2* mRNA in both animal models. No significant changes were observed in the mRNA expression of Mrgprb2 in both animal models (*P* = 0.44 and *P* = 0.06 for detrusor and urothelium respectively, N = 10, Fig. 2) suggesting that Mrgprb2 receptor is present outside of mast cells in the urinary bladder wall. However, *Mrgprb2* mRNA expression is significantly higher in the urothelium in both mouse models (*P* = 0.0028 and *P* = 0.0061 for C57Bl/6 and *C-kit*^*W-sh*^ mice respectively). mRNA expression was normalized to β-actin *(Actb)* expression.

### Effects of Compound 48/80 are mediated through Mrgprb2

**Figure 3:**
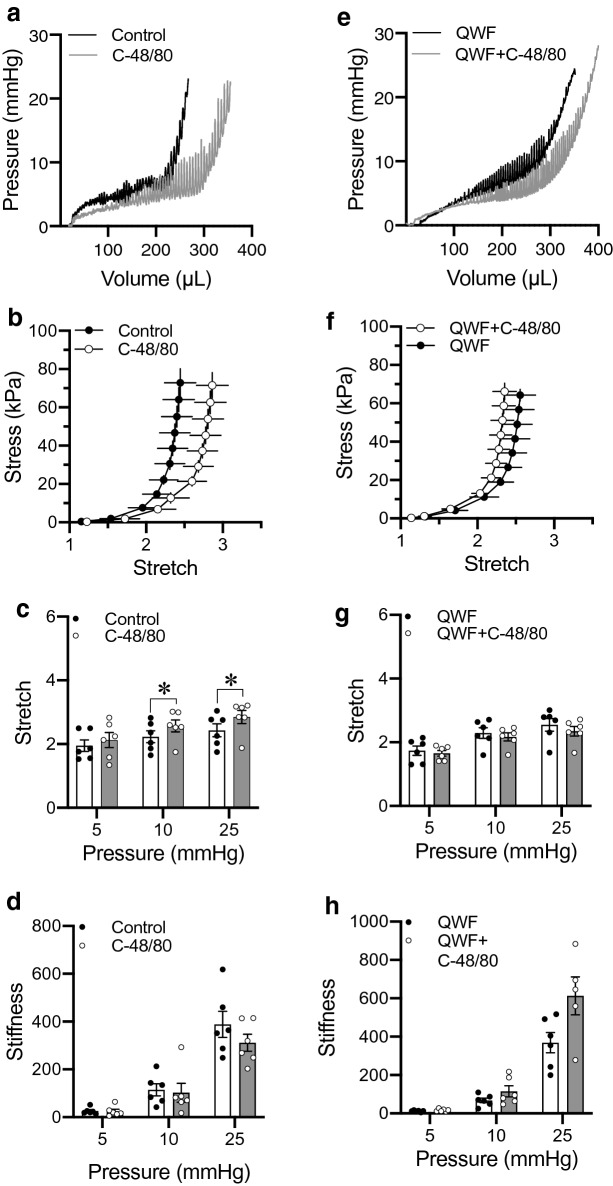
Compound 48/80 increases bladder wall compliance through Mrgprb2 activation. (**a**) Representative pressure–volume trace of ex vivo filling of C57Bl/6 mouse bladders in the absence and presence of the Mrgprb2 agonist Compound 48/80 alone (10 µg/mL). (**e**) representative pressure–volume trace after prior exposure to the Mrgprb2 antagonist QWF (10 µM). (**b**,**c**) compliance is increased in the presence of C-48/80 (N = 6) signified by the rightward shift of the stress-stretch curve and a significant increase in stretch at 10 (*P* = 0.011 vehicle *vs.* C-48/80) and 25 mmHg (*P* = 0.01 vehicle *vs.* C-48/80). (**f**,**g**) the increase in compliance by C-48/80 is blocked by QWF (N = 6) signified by the absence of rightward shift of the stress-stretch curve and increase in stretch (*P* = 0.4, *P* = 0.14 and *P* = 0.07 for 5, 10 and 25 mmHg respectively; QWF *vs.* QWF + C-48/80). (**d**,**h**) Stiffness is unchanged in both groups. Within the group comparisons were made via two-tailed, paired Student’s t-test. “C-48/80”: 10 µg/mL Compound 48/80; “QWF”: 10 µM QWF.

The orphan receptor Mrgprb2 is traditionally believed to be expressed on mast cells, nerve fibers, and keratinocytes, where it mediates pseudo-inflammatory pathways^30^. While traditionally described as an ambiguous mast cell activator, responses to Compound 48/80 and its endogenous analogue Substance P are both mediated by the Mrgprb2 receptor^22,31^. To investigate the effects of Compound 48/80 on wall compliance, we recorded intravesical pressure and pentaplanar images (Fig. 3a; Supplementary Video 1) during ex vivo whole bladder filling using the Pentaplanar Reflected Image Macroscopy (PRIM) System. These data were then used to calculate bladder wall stress and stretch during filling. Wall compliance rapidly increased after exposure to Compound 48/80 (10 µg/mL), as demonstrated by a rightward shift of the stress-stretch curve (Fig. 3b, N = 6) and by the significant increase in stretch at 10 mmHg (*P* = 0.011) and 25 mmHg (*P* = 0.01; Fig. 3c). Wall stiffness remained unchanged after exposure to Compound 48/80 (*P* = 0.91, *P* = 0.58 and *P* = 0.09 for 5, 10 and 25 mmHg respectively; Fig. 3d). To determine if the increase in compliance caused by Compound 48/80 was mediated by Mrgprb2, ex vivo bladder filling was next performed in the absence or presence of the Mrgprb2 antagonist QWF (10 µM) (N = 6, Fig. 3e). Compound 48/80 did not increase wall compliance in the presence of QWF (Fig. 3f), nor did it significantly alter stretch (*P* = 0.40, *P* = 0.140 and *P* = 0.070 for 5, 10 and 25 mmHg respectively; Fig. 3g) or stiffness (*P* = 0.110, *P* = 0.130 and *P* = 0.140 for 5, 10 and 25 mmHg respectively; Fig. 3h) at 5, 10 and 25 mmHg. Neither vehicle nor QWF significantly affected the mechanical properties of the bladder wall in the absence of Compound 48/80 (Supplementary Fig. 1A–D). This suggests that increased wall compliance caused by Compound 48/80 is due to Mrgprb2 activation.

### Effects of Compound 48/80 on bladder compliance are mast cell independent

**Figure 4:**
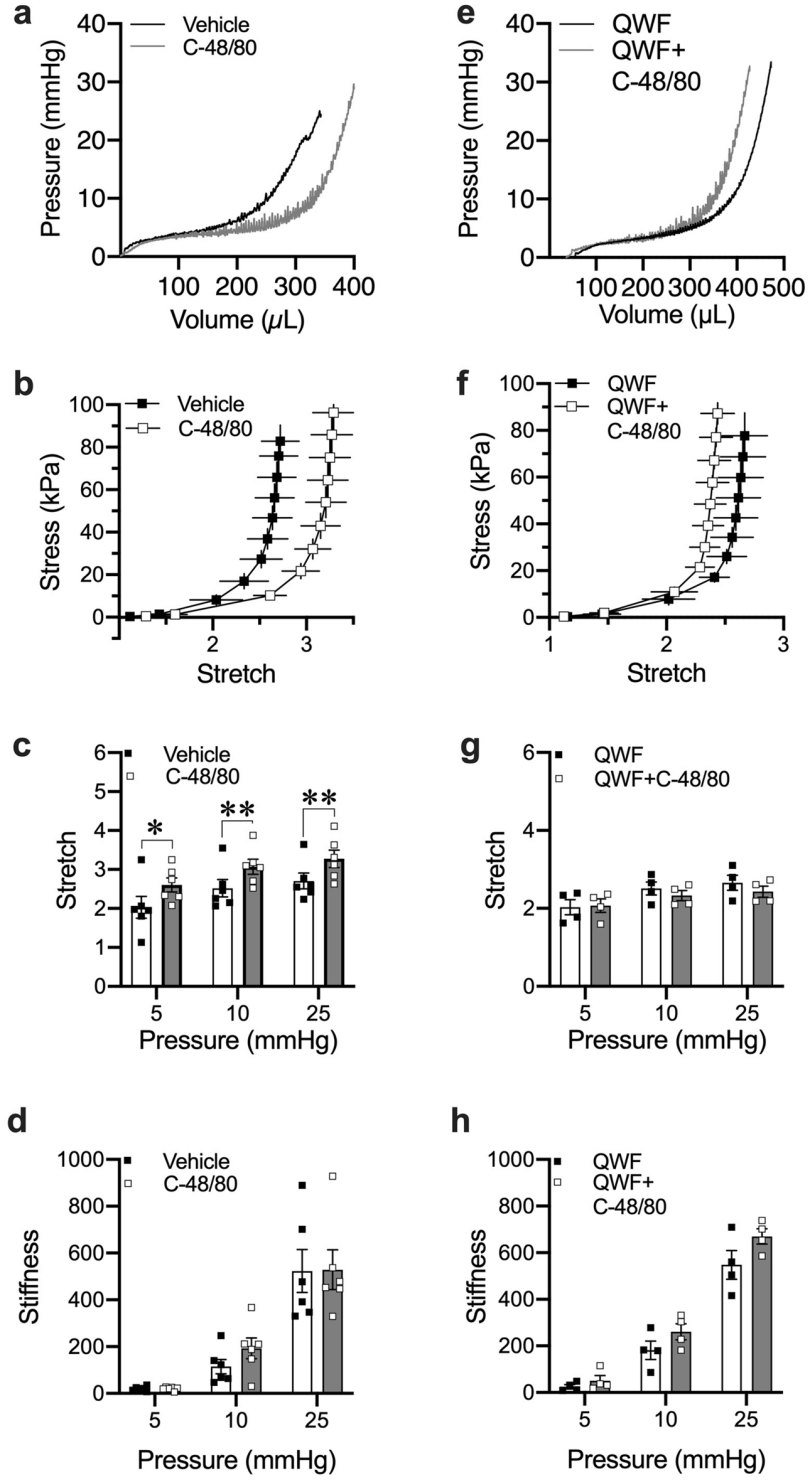
Compound 48/80 increases bladder wall compliance independent of mast cells. (**a**) Representative pressure–volume trace of ex vivo filling of *C-Kit*^*W-Sh*^ mouse bladders in the absence and presence of the Mrgprb2 agonist Compound 48/80 alone (10 µg/mL) (**e**) representative pressure–volume trace after prior exposure to the Mrgprb2 antagonist QWF (10 µM). (**b**,**c**) As with C57Bl/6 mice, compliance is increased in the presence of C-48/80 in mast cell-deficient *C-Kit*^*W-Sh*^ mice (N = 6) signified by the increase in stretch (*P* = 0.01, *P* = 0.006 and *P* = 0.008 for vehicle *vs.* C-48/80) at 5, 10, and 25 mmHg respectively. (**f**,**g**) The increase in compliance by C-48/80 was again blocked by QWF (N = 4) signified by the overlapping stress-stretch curves and no increase in stretch *(P* = 0.343, *P* = 0.255 and *P* = 0.282 for 5, 10 and 25 mmHg respectively for QWF *vs.* QWF + C-48/80). (**d**,**h**) similar to wild-type, stiffness remains unchanged. Within the group comparisons were made via two-tailed, paired Student’s t-test. “C-48/80”: 10 µg/mL Compound 48/80; “QWF”: 10 µM QWF.

Mice with the *C-kit*^*W-sh*^ mutation do not express mast cells^32^. To determine if Compound 48/80 increases bladder compliance in a mast cell-mediated manner, ex vivo filling was again performed in the PRIM system using bladders from mast cell-deficient *C-kit*^*W-sh*^ mice (N = 6). *C-kit*^*W-sh*^ mice showed no significant differences compared to wild-type mice in mechanical compliance, stretch, or stiffness in the absence of Compound 48/80 (Supplementary Fig. 2A–C). Compound 48/80 still caused a rightward shift in the stress/stretch curve and increased bladder wall stretch at 5 mmHg (*P* = 0.010), 10 mmHg (*P* = 0.006) and 25 mmHg (*P* = 0.008) (Fig. 4a–c). Similar to wild-type mice, stiffness was also unchanged at all pressures (*P* = 0.960, *P* = 0.183 and *P* = 0.958 for 5, 10 and 25 mmHg respectively; Fig. 4d). The increase in stretch was prevented by QWF (*P* = 0.343, *P* = 0.255 and *P* = 0.282 at 5, 10 and 25 mmHg respectively, N = 4, Fig. 4e–g). There was no change in stiffness after prior exposure to QWF (*P* = 0.124, *P* = 0.340 and *P* = 0.293 at 5, 10 and 25 mmHg respectively, N = 4, Fig. 4h). Similar to wild-type mice, neither vehicle nor QWF significantly affected the mechanical properties of the bladder wall in the absence of Compound 48/80 (Supplementary Fig. 3A–D). Together, these results suggest that the effects of Compound 48/80 on urinary bladder wall compliance are independent of mast cells but dependent on Mrgprb2.

### Compound 48/80 increases clinical compliance

**Figure 5:**
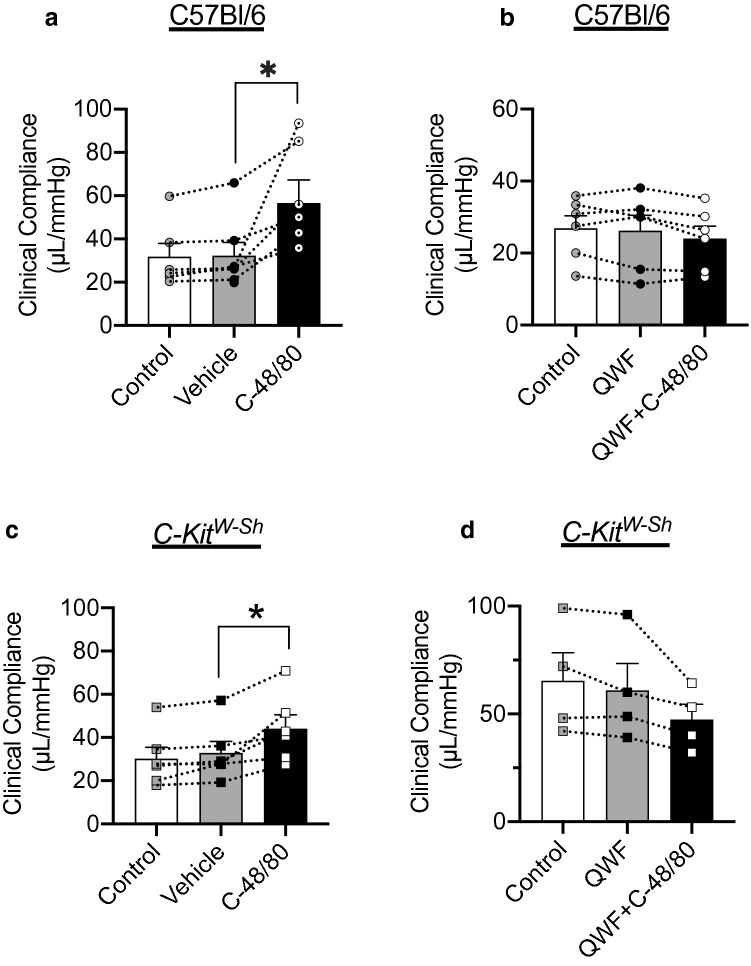
Compound 48/80 significantly increases clinical compliance independent of mast cell activation. (**a**,**c**) Clinical compliance was increased after exposure to 10 µg/mL Compound 48/80 in both C57Bl/6 (*P* = 0.01; N = 6, C-48/80 *vs*. Vehicle) and *C-kit*^*W-Sh*^ mice (*P* = 0.034; N = 6, C-48/80 *vs.* Vehicle). (**b**,**d**) this increase was blocked by 10 µM QWF in both C57Bl/6 (P = 0.206; N = 6, QWF *vs.* QWF + C-48/80) and *C-kit*^*W-Sh*^ mice (P = 0.209; N = 4, QWF *vs.* QWF + C-48/80). No significant differences were seen between control and vehicle in all groups. Within the group comparisons were made via one-way ANOVA followed by Tukey’s *post-hoc* analysis. “C-48/80”: 10 µg/mL Compound 48/80; “QWF”: 10 µM QWF.

As a comparator to our measures of stretch and stress, we also calculated clinical compliance in terms of the change in volume per change in pressure during the quasi-linear portion of bladder filling*.* In the absence of Compound 48/80, no differences in clinical compliance were seen between C57Bl/6 and *C-Kit*^*W-Sh*^ mice (Supplementary Fig. 2D). Compound 48/80 increased clinical compliance in wild-type (*P* = 0.01, N = 6, Vehicle *vs.* C-48/80; Fig. 5a) and *C-Kit*^*W-Sh*^ mice (*P* = 0.034, N = 6, Vehicle *vs.* C-48/80; Fig. 5c). In both mouse models, the increase was blocked by QWF (*P* = 0.206, N = 6, Fig. 5b and P = 0.209, N = 4, Fig. 5d).

### Activation of Mrgprb2 by Compound 48/80 increases UBSM Excitability

**Figure 6:**
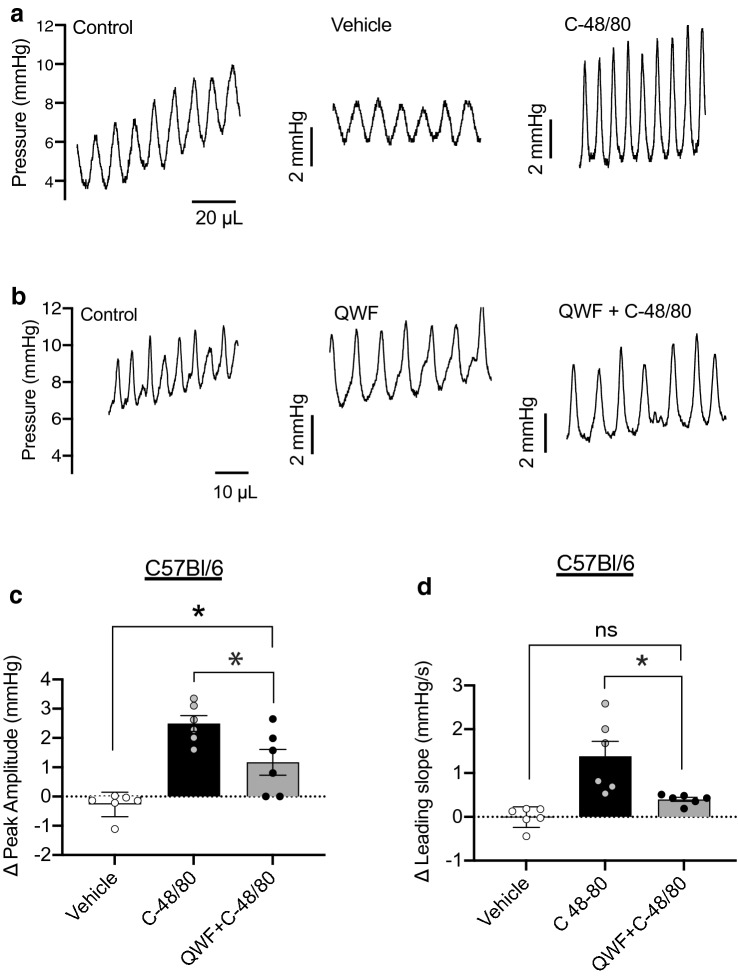
Compound 48/80 increases the amplitude and leading slope of transient contractions through Mrgprb2 activation. Representative trace of transient pressure events during ex vivo filling of a C57Bl/6 mouse bladder during control fill, fill in presence of vehicle (**a**)  10 µM QWF (**b**) (N = 6), and after exposure to 10 µg/mL Compound 48/80 (N = 6). (**c**,**d**) the increase in both leading slope (*P* = 0.014, N = 6, C-48/80 *vs.* QWF + C-48/80) and peak amplitude (*P* = 0.024, N = 6, C-48/80 *vs.* QWF + C-48/80) by Compound 48/80 was blocked by QWF. There was an increase in peak amplitude due to Compound 48/80 even in the presence of QWF (*P* = 0.014 Vehicle *vs.* QWF + C-48/80, Fig. 6c). No significant differences in leading slope and peak amplitude were seen in the presence of QWF alone. Between group comparisons were made via one-way ANOVA followed by Tukey’s *post-hoc* analysis. “C-48/80”: 10 µg/mL Compound 48/80; “QWF”: 10 µM QWF.

**Figure 7:**
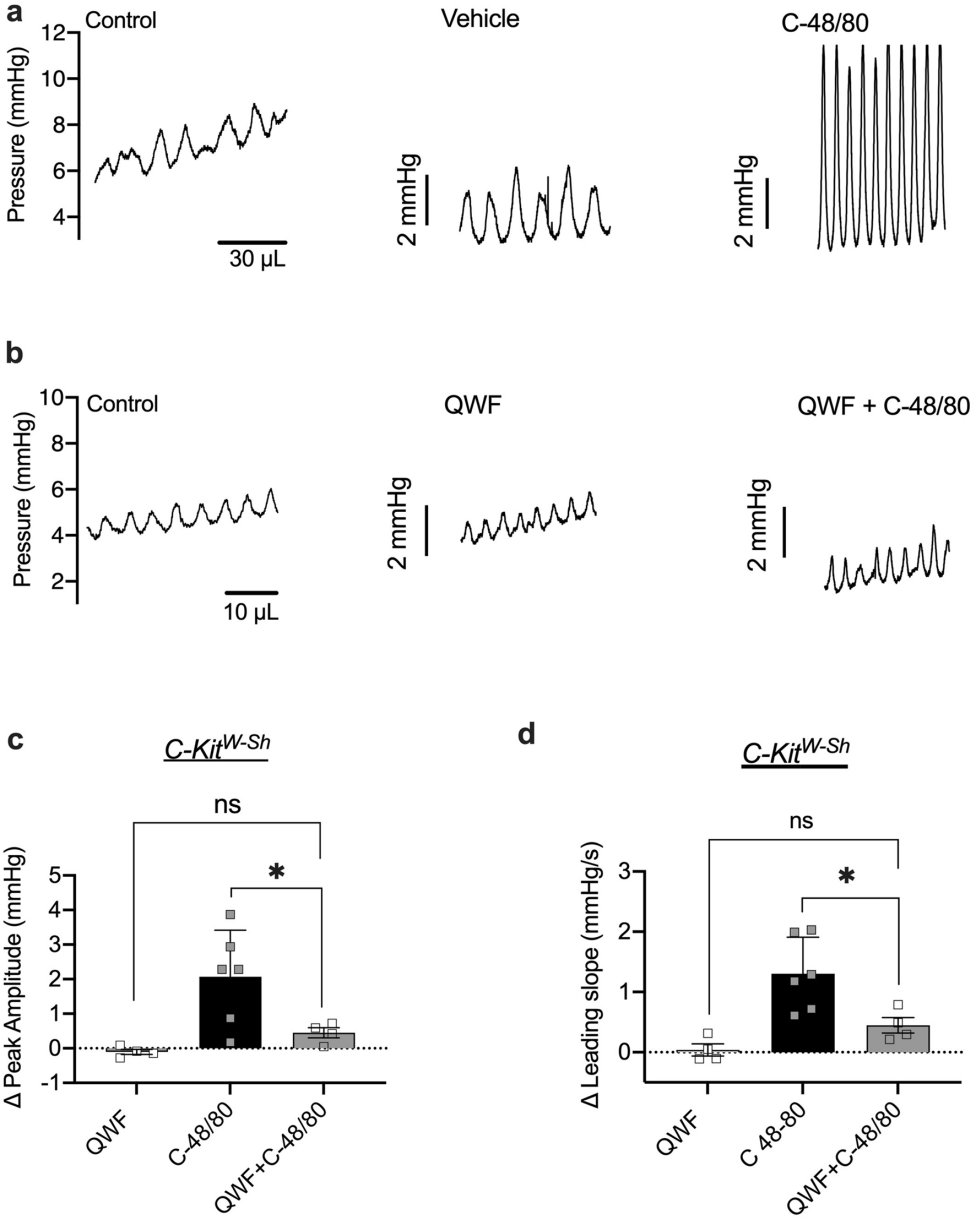
Compound 48/80 increases the amplitude and leading slope of transient contractions independent of mast cell activation. Representative trace of transient pressure events during ex vivo filling of a *C-kit*^*W-Sh*^ mouse bladder during control fill, fill in presence of vehicle (**a**) 10 µM QWF (**b**) (N = 4) and after exposure to 10 mg/mL Compound 48/80 (N = 6). (**c**,**d**) The increase in both leading slope (*P* = 0.030) and peak amplitude (*P* = 0.048) by Compound 48/80 was blocked by QWF. No significant differences in leading slope and peak amplitude were seen in the presence of QWF alone. Between group comparisons were made via one-way ANOVA followed by Tukey’s *post-hoc* analysis. “C-48/80”: 10 µg/mL Compound 48/80; “QWF”: 10 µM QWF**.**

Transient pressure events are responsible for driving sensory outflow to the central nervous system during filling, and their rate of rise directly correlates with the amplitude of afferent bursts^15^. Thus, we analyzed the amplitude and rate of rise of transient pressure events as a measure of UBSM excitability as well as a surrogate measure of associated sensory outflow. Compound 48/80 significantly increased the amplitude and the rate of rise of transient contractions as compared to vehicle in both mouse models (Figs. 6a and  7a). The effect of Compound 48/80 on peak amplitude and leading slope was reduced by QWF in wild-type mice (*P* = 0.024 and *P* = 0.010 for 6C and 6D respectively, QWF + C-48/80 vs. C-48/80, N = 6, Fig. 6a–d). There was an increase in peak amplitude due to Compound 48/80 even in the presence of QWF, but it was significantly smaller than Compound 48/80 alone (*P* = 0.014 Vehicle vs. QWF + C-48/80, Fig. 6c). Similar to wild-type mice, effects of Compound 48/80 were reduced by QWF in mast cell deficient mice (*P* = 0.048; N = 6 and *P* = 0.03 for 7C and 7D respectively, QWF + C-48/80 vs. C-48/80, Fig. 7a–d). Collectively, these findings indicate that while the bladder is more compliant after exposure to Compound 48/80, UBSM excitability (and possibly afferent outflow) are also increased. These findings also suggested that the effects of Compound 48/80 on bladder wall compliance and transient pressure event leading slope are mediated by Mrgprb2.

### Treatment with Compound 48/80 causes bladder overactivity

**Figure 8:**
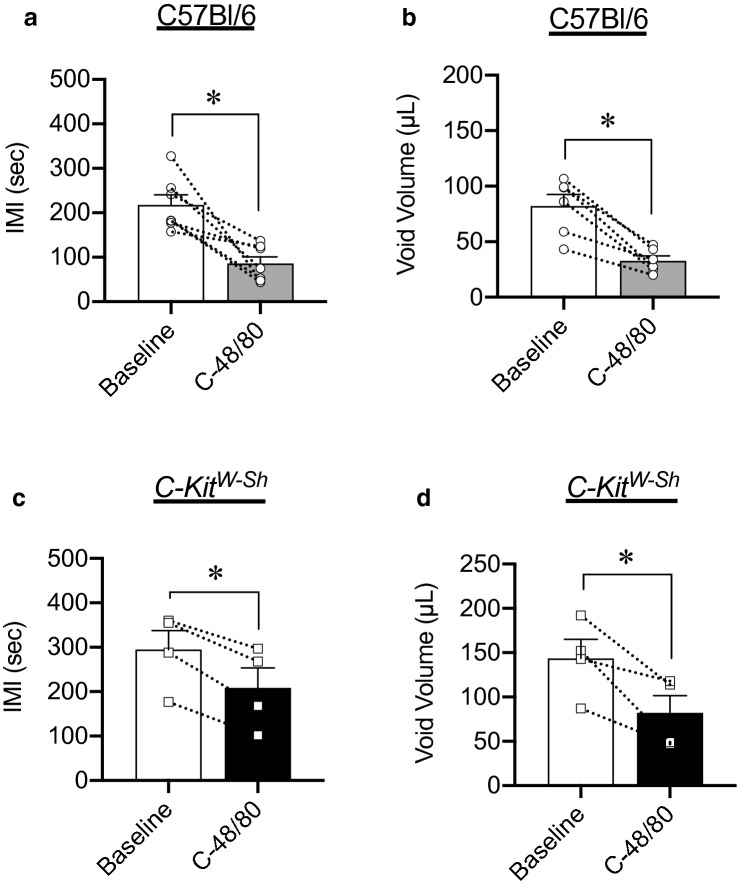
Compound 48/80 decreases causes bladder overactivity in wild-type and mast cell-deficient mice. Intravesical instillation of Compound 48/80 (50 µg/mL) significantly decreased intermicturition interval (**a**) (*P* = 0.005) and void volume (**b**) (*P* = 0.002) in C57Bl/6 mice (N = 8) during conscious cystometry. (**c**,**d**) Compound 48/80 had the same effect on intermicturition interval and void volume in mast cell-deficient *C-kit*^*W-Sh*^ mice (*P* = 0.005 and *P* = 0.04, N = 4). Within the group comparisons were made via paired Student’s t-test.

We next determined if the ex vivo effects of Compound 48/80 translated into in vivo changes in urodynamics. In order to prevent systemic activation of mast cells, conscious cystometry was performed before and after intravesical infusion of Compound 48/80. In order to overcome the permeability barrier formed by umbrella cells lining the bladder without inducing inflammation or destroying the integrity of the urothelium, a higher concentration of Compound 48/80 (50 µg/mL) was used in these experiments. Compound 48/80 decreased intermicturition interval (IMI) and void volume in C57Bl/6 mice, indicative of bladder overactivity (*P* = 0.005 and *P* = 0.002 for IMI and void volume respectively, N = 8, Fig. 8a,b). These findings were recapitulated in *C-kit*^*W-Sh*^ mice, again suggesting that the effects of Compound 48/80 were mast cell-independent (*P* = 0.005 and *P* = 0.040 for IMI and void volume respectively, N = 4, Fig. 8c,d).

## Discussion

Using cystometry, knockout mouse models, gene expression assays, and the novel pentaplanar reflected image macroscopy (PRIM) system, we comprehensively assessed how the mast cell activator Compound 48/80 alters urinary bladder function. While Compound 48/80 increased urination frequency in vivo and increased transient pressure events and mechanical compliance ex vivo, it did so in a mast cell-independent manner. The effects of Compound 48/80 were instead mediated by Mrgprb2 receptors presumably expressed in other cells within the urinary bladder wall itself. One interesting finding of this study was the speed at which Compound 48/80 can cause profound increases in urinary bladder compliance: within 30 min of exposure, both bladder wall mechanical compliance and UBSM excitability were augmented. These data suggest that: (1) basic secretagogues can alter bladder function independent of traditional mast cell-mediated and nerve-mediated pathways; and (2) rapid increases in bladder compliance correlate with bladder overactivity, likely related to increased sensory outflow during transient contractions of the bladder wall. Our findings also suggest that the Mrgprb2 receptor is an important linchpin in regulating bladder wall remodeling and changes to UBSM excitability caused by inflammation.

### Clinical versus mechanical compliance

Many previous studies use “compliance” as a measure of stiffness and elasticity during filling^33^. However, the term “compliance” is often inaccurately defined^34^. Clinically, bladder compliance is calculated as the change in volume per change in intravesical pressure^35^. This definition, however, is incomplete as it disregards any variations in wall structure and thickness that occur during filling. As such, the traditional measure of clinical compliance would only be accurate if every bladder is the same size and thickness. By omitting the amount of bladder wall present and the contribution of each constituent of the bladder wall’s structure, this definition significantly skews our understanding of how the bladder wall remodels in disease. Clinical compliance also gives incomplete information about changes is the bladder wall structure and content itself, as it cannot differentiate between geometric stiffness (stiffness imparted due to the shape of the bladder) and mechanical stiffness (stiffness intrinsic to the material properties of the bladder wall). Instead, it only considers how both of these together influence the static second phase of bladder filling where pressure increases the least^36^. In this study, we instead measure bladder compliance as the relationship between wall stress and stretch. These factors consider the distribution of force across the total amount of bladder wall as it fills^6^ and account for the volume of wall present in each bladder. Thus, accurate and appropriate comparisons of bladder wall mechanical properties can be made between subjects. We also included measures of clinical compliance as a means of comparing “traditional” measures of compliance with our calculations of stress, stretch, and stiffness. Even though our conclusions from our experiments were similar in each methodology, the ability to determine changes in stretch and stiffness at multiple points during filling brings information impossible to glean from pressure and volume alone.

### Effects of Compound 48/80 on the urinary bladder function

Compound 48/80 has been extensively studied as a mast cell degranulating compound in the bladder as well as other organ systems^37–39^. Previous work utilizing Compound 48/80 to study the effects of mast cell-driven inflammation showed heightened UBSM excitability indirectly due to effects on peripheral endings of primary afferent neurons^40^. None of these studies considered the effects Compound 48/80 may have on the mechanical properties of the bladder wall and if changes to these properties are mast cell dependent. This study is the first of its kind to show that an increase in mechanical compliance accompanies increased UBSM contractility after exposure to Compound 48/80. These effects suggest that the immediate response to inflammation in the bladder is both an increase in contractility along with a paradoxical increase in compliance, which can happen within minutes and lead to bladder overactivity. These results were counterintuitive since bladder overactivity is most often associated with a decrease in wall compliance and an increased muscle tone^9,41^. Our findings instead suggest that the first step in bladder wall remodeling is an initial loss of ECM interconnectivity that increases distensibility, which is perhaps then followed by deposition of improperly arranged collagen that leads to remodeling. Such deposition is noted in rat models of overactive bladder due to spinal cord injury, where changes in collagen tortuosity and morphology increase bladder distensibility^42^.

Interestingly, the change in compliance is almost entirely due to increased bladder wall stretch. Wall stiffness remained unchanged, implying that while the arrangement and interconnections of the ECM change acutely in response to Compound 48/80, the mechanical properties of the constituent elements remain the same. These findings also suggest that the effect of muscle tone on wall compliance may have been overestimated in the past^43^. Thus, the structural arrangement of the extracellular matrix may have a stronger influence on wall mechanics than muscle tone when considering the distribution of forces during the entirety of bladder filling.

The most interesting and surprising finding in this study was that effects of Compound 48/80 persisted in the absence of mast cells. Compound 48/80, a prototypical mast cell activator, degranulates mast cells by acting on the orphan Mrgprb2 receptor present in the mast cell membrane^44^. Our findings support the idea that Mrgprb2 is not mast cell specific, but instead is expressed throughout the bladder with the highest expression being in the urothelial layer. Furthermore, the effects of Compound 48/80 on both compliance and UBSM contractility imply that activation of Mrgprb2 may cause release of inflammatory mediators normally attributed to mast cell degranulation irrespective of whether mast cells are present or not. When released from mast cells, mediators alter both the organization of the extracellular matrix and UBSM activity^45^ and lead to effects on wall compliance and transient contractions. Thus, it appears that the presence of Mrgprb2 in other bladder cells causes them to behave like quasi-immune cells to drive remodeling and UBSM hyperexcitability.

### Function and pharmacology of Mas-related G protein-coupled receptor B2

Mrgprb2 (the mouse ortholog of human MRGPRX2) is an orphan receptor primarily studied in the context of non-IgE mediated mast cell degranulation and inflammation^46,47^. Activation of MRGPRX2 and Mrgprb2 using basic secretagogues and neurokinins brings about IgE-independent anaphylaxis^48^. Transcripts from both MRGPRX2 and Mrgprb2 were detected in skin, sensory neurons, urinary bladder, and adipose tissue, though the presence of mast cells in these tissues was not ruled out as the source of Mrgprb2/X2 mRNA^49–52^. While prior research has primarily focused on the skin in context with MRGPRX2-dependent reactions, adult urinary bladder has the third highest level of Mrgprb2/X2 mRNA of all tissues surveyed^53^. Therefore, effects of Mrgprb2 on the components of the bladder wall (urothelial cells, UBSM, extracellular matrix, etc.) are of potential interest in terms of lower urinary tract dysfunction. Our study piques this interest even more, as it is the first to demonstrate the mast cell-independent effects of Mrgprb2 activation specifically in the bladder ex vivo and in vivo*.* Our findings also suggest that *Mrgprb2* mRNA is predominantly expressed in the urothelium. This was not completely surprising since the urothelium is also a transitional epithelium that may act like secretory cells upon activation of inflammatory pathways, very much like keratinocytes^54,^ which are an identified cell type that express Mrgprb2 receptors. Thus, Mrgprb2 receptors may be key in the development of bladder dysfunction associated with inflammatory insult and fibrosis.

Due to the dual nature of its actions, the pathway (or pathways) that Compound 48/80 activates may be two-fold and parallel to each other. Hypothetically, Mrgprb2 receptors present on urothelial cells could make them respond to basic secretagogues like immune cells—specifically mast cells. This is not unprecedented, since urothelial cells produce and release multiple products common to mast cell degranulation that increase UBSM contractility^55^. At the same time, activation of Mrgprb2 could also release mediators that activate downstream pro-inflammatory pathways, including the release and activation of matrix metalloproteases, that alter the structure and organization of the extracellular matrix^11,56^. The nature of this dual effect may make the pathway of Mrgprb2 activation in the bladder extremely significant in both health and disease. Based on our findings and assuming similar expression patterns in humans, targeting human MRGPRX2 receptors could alleviate both the increased excitability and remodeling intrinsic to LUTS without preventing UBSM contractions in response to parasympathetic stimuli.

The lack of specific compounds that activate or inhibit Mrgprb2, however, poses an obstacle in receptor specificity studies. Some cationic peptides similar to Compound 48/80 activate Mrgprb2 and produce a local edema accompanied by itch when given subcutaneously^48^. Neurokinin-1 (NK_1_) receptor antagonists show efficacy in animal models, but are inactive on human MRGPRX2 except QWF^23^. Compound 48/80 remains the only diagnostic compound that is a highly potent Mrgprb2/X2 agonist. Similarly for antagonists, QWF is the only compound that purports to be an antagonist for both Mrgprb2 and MRGPRX2. The generation of more selective and potent drugs will markedly aid in our understanding of how and where these receptors drive physiology.

Our initial hypothesis stated that the increase in UBSM activity and wall compliance is brought about by activation of Mrgprb2 since the effects of Compound 48/80 were inhibited by the Mrgprb2 antagonist QWF. QWF also inhibits NK_1_ receptors, thus the activation of NK_1_ receptors by Compound 48/80 was still a possibility. Itch responses evoked by Mrgprb2 activation are inhibited by QWF even in the absence of NK_1_ receptors^23^, suggesting that although QWF antagonizes both receptors, the effects of Compound 48/80 are likely only mediated by Mrgprb2. This complexity means that specific actions of Compound 48/80 associated with Mrgprb2 activation cannot be clearly delineated by pharmacology alone in whole bladder tissue. Future projects will focus on developing specific and potent modulators of Mrgprb2. Alternatively, NK_1_ receptor knockout mice could be used to perform similar stress-stretch analysis studies to confirm the effects of both Compound 48/80 and QWF in the bladder depend on Mrgprb2.

## Conclusions

In conclusion, activation of the orphan receptor Mrgprb2 by Compound 48/80 alters the true mechanical properties of the bladder wall independent of mast cells by rapid rearrangement of the bladder wall matrix without changing the inherent mechanical stiffness of the constituent elements of the wall. The present study also demonstrates that muscle tone and excitability have surprisingly little influence on wall compliance, as Compound 48/80 increased wall compliance even in the face of an increase in UBSM excitability. This suggests that bladder wall structure and mechanical properties are more labile than originally thought, and rapidly influenced by the actions of basic secretagogues acting on Mrgprb2. Future studies will determine the exact location of Mrgprb2 in the urinary bladder and assess the mediators released upon its activation.

## Supplementary Information

Supplementary Information.

## Data Availability

The datasets generated and/or analyzed during the current study are not publicly available but are available from the corresponding author on reasonable request.
