# Supplementary material for: Compound 48/80 increases murine bladder wall compliance independent of mast cells
Source: Sci Rep. 2023 Jan 12;13:625. doi: 10.1038/s41598-023-27897-6 (PMC9837046; doi:10.1038/s41598-023-27897-6)
Supplement: Supplementary file 1 — Supplementary Information. [file 41598_2023_27897_MOESM1_ESM.pptx]

## Slide 1
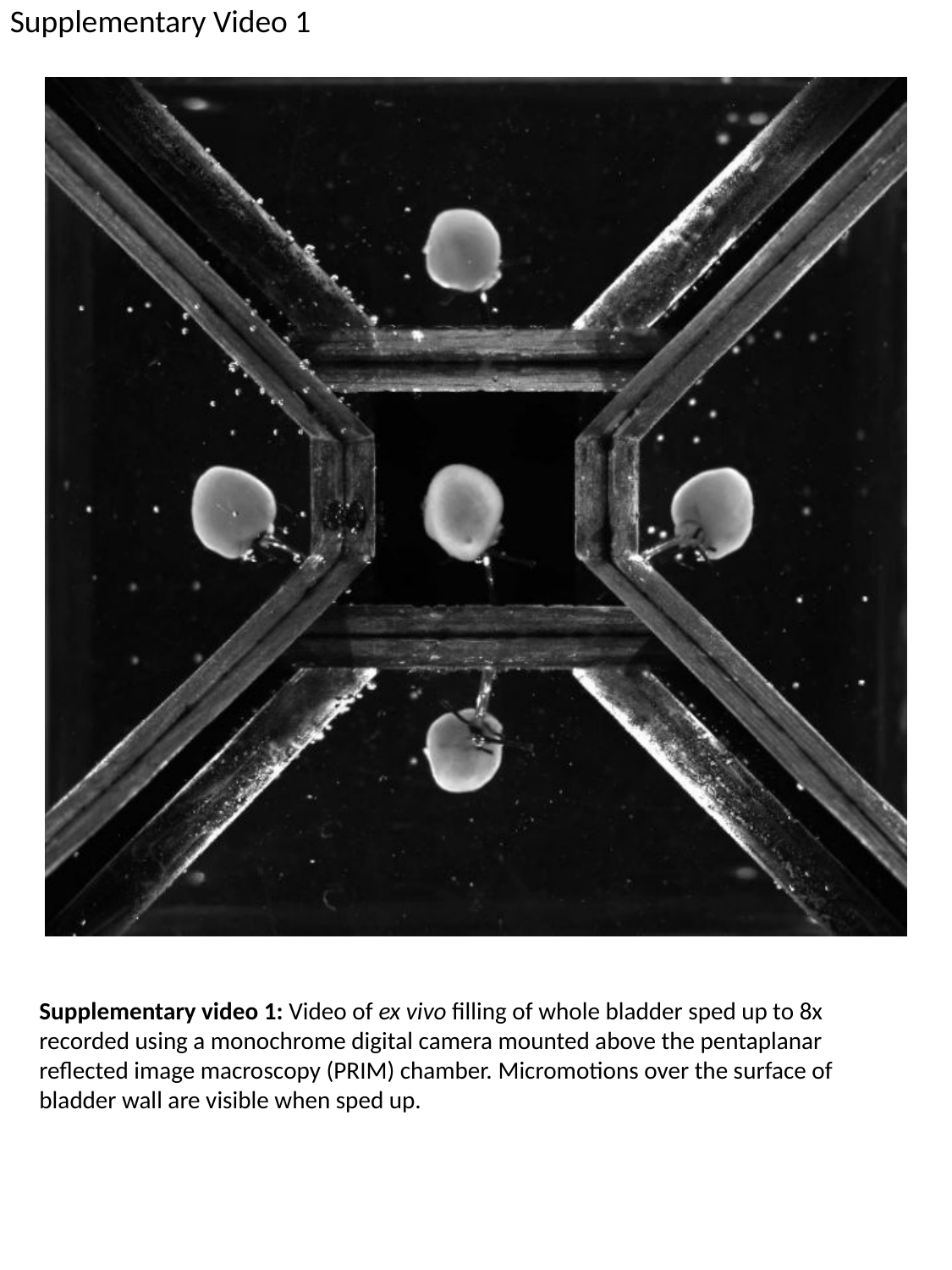

Supplementary Video 1
Supplementary video 1: Video of ex vivo filling of whole bladder sped up to 8x recorded using a monochrome digital camera mounted above the pentaplanar reflected image macroscopy (PRIM) chamber. Micromotions over the surface of bladder wall are visible when sped up.

## Slide 2
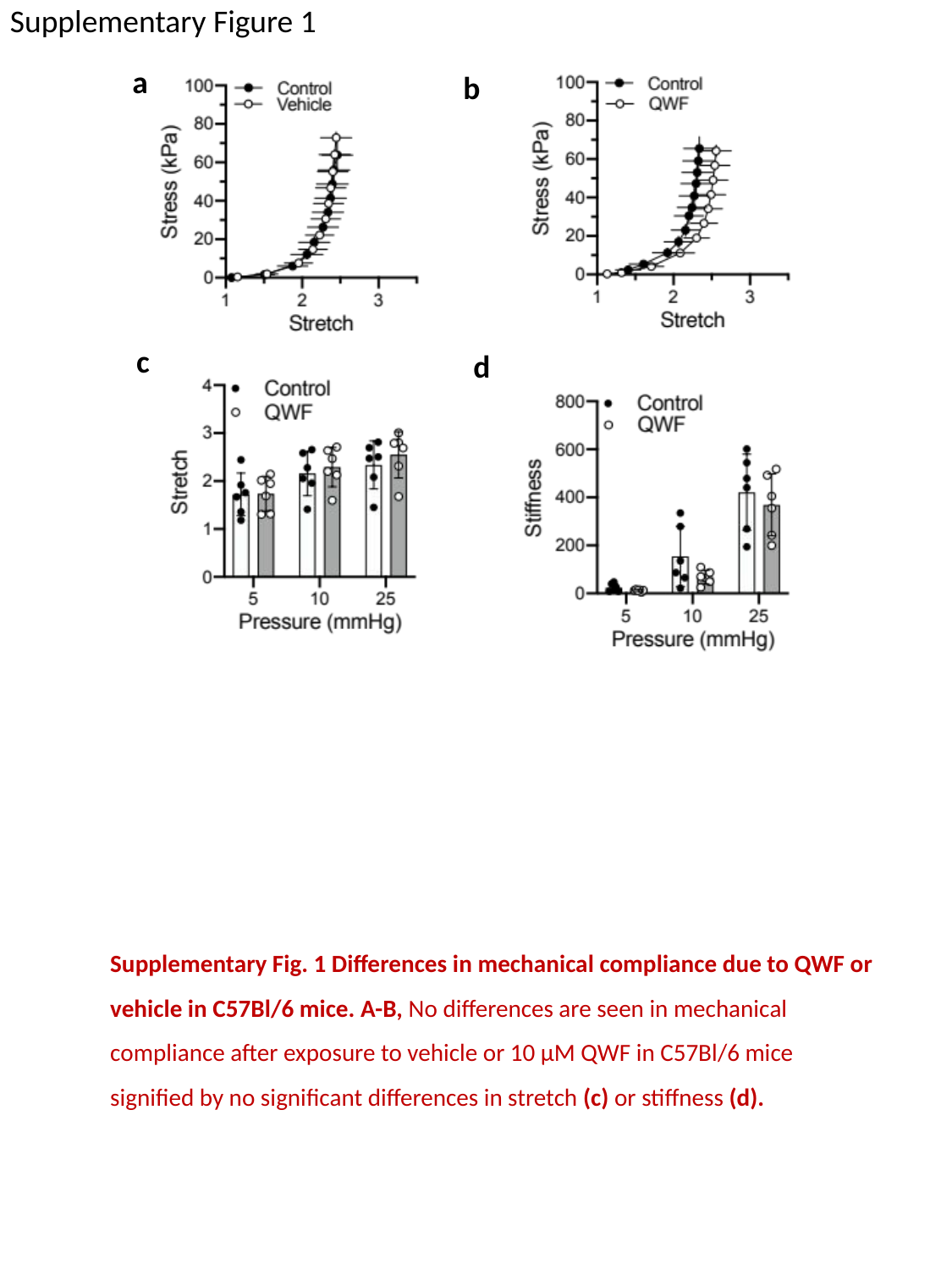

Supplementary Figure 1
a
b
c
d
Supplementary Fig. 1 Differences in mechanical compliance due to QWF or vehicle in C57Bl/6 mice. A-B, No differences are seen in mechanical compliance after exposure to vehicle or 10 µM QWF in C57Bl/6 mice signified by no significant differences in stretch (c) or stiffness (d).

## Slide 3
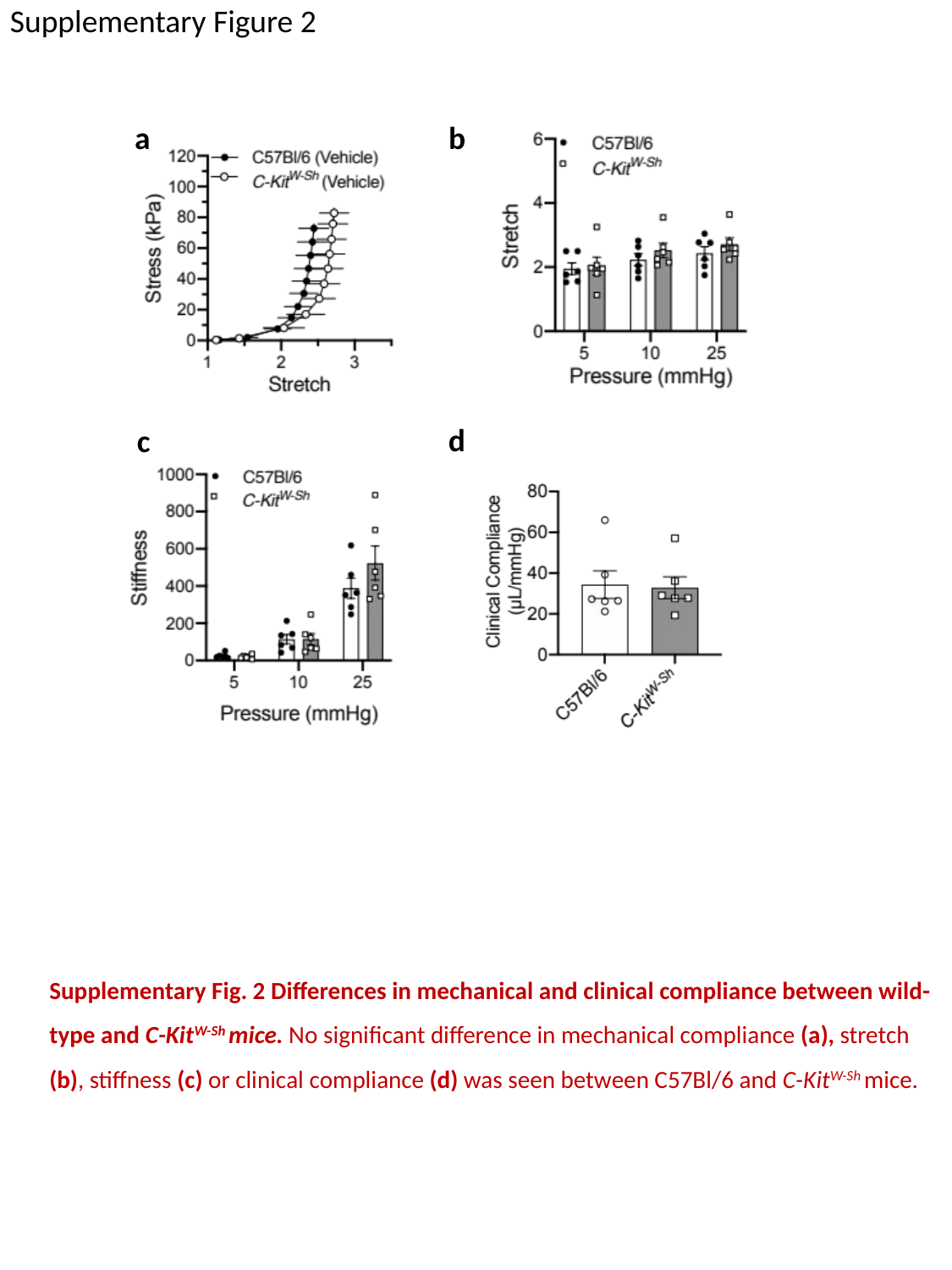

Supplementary Figure 2
a
b
d
c
Supplementary Fig. 2 Differences in mechanical and clinical compliance between wild-type and C-KitW-Sh mice. No significant difference in mechanical compliance (a), stretch (b), stiffness (c) or clinical compliance (d) was seen between C57Bl/6 and C-KitW-Sh mice.

## Slide 4
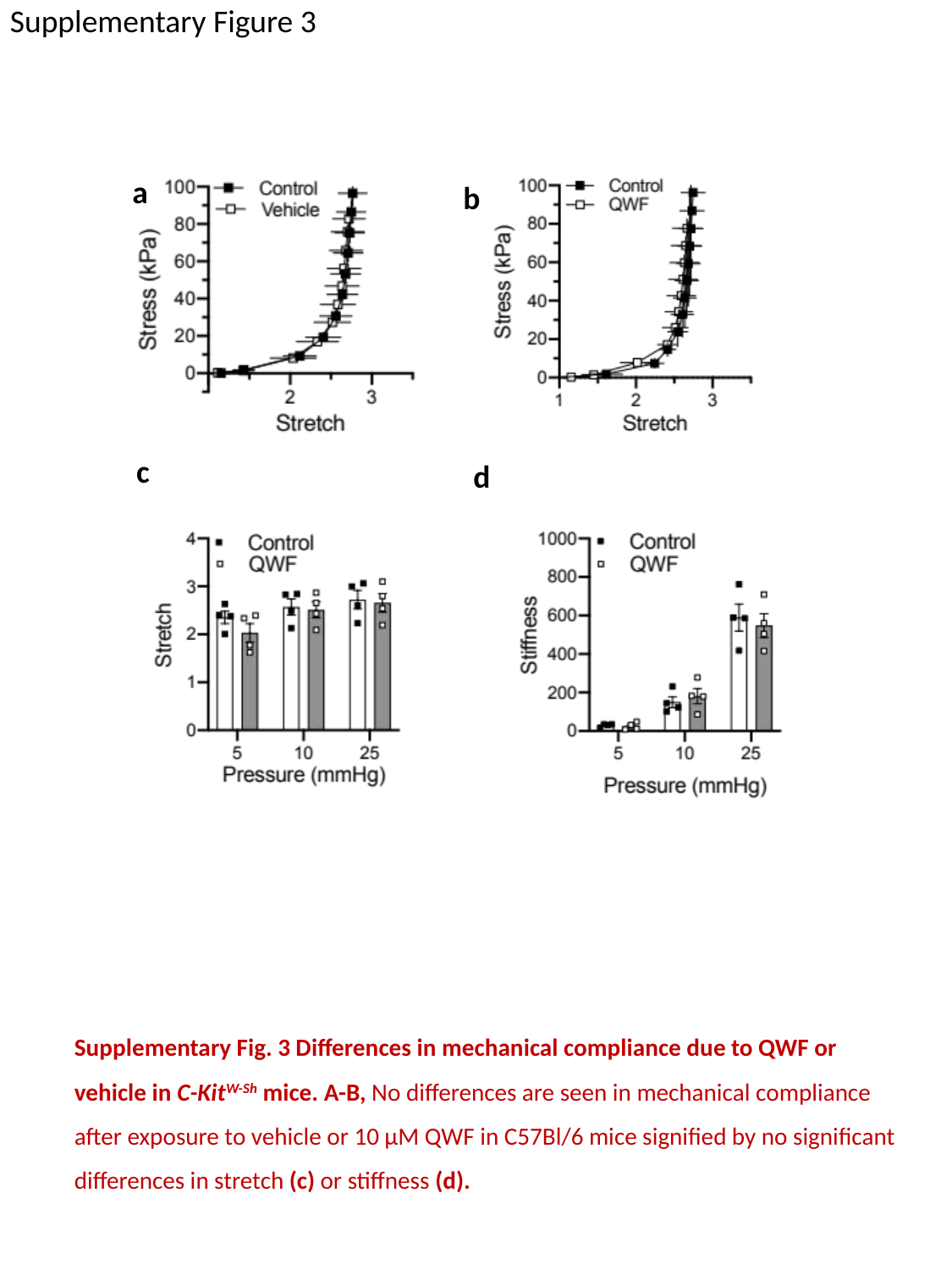

Supplementary Figure 3
a
b
c
d
Supplementary Fig. 3 Differences in mechanical compliance due to QWF or vehicle in C-KitW-Sh mice. A-B, No differences are seen in mechanical compliance after exposure to vehicle or 10 µM QWF in C57Bl/6 mice signified by no significant differences in stretch (c) or stiffness (d).
